# Challenges with international medical graduate selection: finding positive attributes predictive of success in family medicine residency

**DOI:** 10.1186/s12875-022-01861-1

**Published:** 2022-09-29

**Authors:** Alasdair Nazerali-Maitland, Christina Douglas

**Affiliations:** 1 Department of Medicine, University of British Columbia, Diamond Healthcare Centre 2775 Laurel Street, Vancouver, BC V5Z 1M9 Canada; 2 University of British Columbia, Vancouver, Canada

**Keywords:** Professionalism, International medical graduate, Selection, Family medicine, Residency

## Abstract

**Background:**

Criteria to select residents most likely to succeed, other than proficiency of their medical knowledge, is a challenge facing preceptors. International Medical Graduates (IMGs) play an integral role in mitigating the high demand for family medicine physicians across Canada. Thus, selecting IMG candidates that have a high probability of succeeding in Canadian educational settings is important. The purpose of this study is to elucidate qualitative attributes that positively correspond to success in residency, to ultimately assist in the selection of IMG residents most likely to achieve family medicine residency.

**Methods:**

Interviews of 13 family medicine preceptors from some of the largest IMG training sites in Canada were performed to collect original data. The data was coded in tandem sequences using standardized coding techniques to increase robustness of results.

**Results:**

The identified positive predictors of an IMG residents’ success are: presence of a positive attitude, proficient communication skills, high level of clinical knowledge, trainability.

**Conclusions:**

The results provide adequate guidelines to assist in selection of IMG residents. Canada is a unique sociocultural setting where standardized selection methods of IMGs have not been employed. By selecting IMG residents who possess these attributes upon inception of residency, benefits of instruction will be maximized and result in residents developing increased aptitudes for patient care.

## Background

International Medical Graduates (IMGs) comprise 8% of the total physicians matched to residency programs in Canada [1]. IMGs are commonly compared to Canadian Medical Graduates (CMGs) and IMGs consistently score lower than CMGs on examinations. Standardized examinations commonly used to compare family medicine residents include: In Training Evaluation Reports (ITERs) and Certification of College of Family Physicians (CCFP) examinations [1]. Despite institutions’ efforts to mitigate this discrepancy shortcomings of IMGs on family medicine examinations is concerning to medical educators [2]. Preceptors are faced with a surplus of IMG candidates, however they consistently fail to select candidates who succeed on examinations in the same proportions as CMGs [1].

The percentage of IMGs in the workforce is approximately 25% and can reach as high as 50% in underserved rural areas [3]. It has been established that the needs of IMGs when entering practice in family medicine are unique to the needs of CMGs and this can be attributed to a variety of reasons. For example, only 60–70% of IMGs entering the workforce are not recent medical graduates [4]. An increasing percentage of IMGs have completed their medical degrees 5–9 years before beginning residency [5]. This gap in training could result in reduced recency of knowledge acquired in medical school and could play a role in IMGs struggling in examinations.

These factors can be partially to blame for failure of many IMGs on family medicine examinations, however this study sought to increase the robustness of selection to better determine candidates who will succeed in Canadian educational programs. The literature has limited exploration of qualitative predictors of IMG performance on family medicine examinations and further replication and expansion of results is implored and was a focus in this study [6].

Qualitative predictors include subjectively assessed attributes possessed by residents. It has been established that IMGs face unique barriers compared to CMGs, selecting for positive attributes should not be used to replace efforts to mitigate these barriers [7]. However, by establishing a list of attributes, which positively correspond to IMG resident success, perhaps candidates can be selected that are better suited for the established education system. Literature reviews have established competency frameworks for IMG physicians, which present roles that underpin the framework of this study [8]. In addition, the CanMEDS framework was a guiding tool for this study as we sought to reproduce these competencies in relation to IMGs’ success in family medicine in Canada, an inquiry which has not been presented in the literature previously [9]. There are many knowledge gaps in the literature with regards to IMG research including selection criteria [10].

The list of attributes presented in this study can be extended to elucidate factors causing a resident to struggle and assist in remediating issues through developing targeted training. IMG needs are not well defined, as they differ vastly depending on the resident and their background [11]. Overall, by assessing these proposed attributes during selection, more robust candidates can be selected and issues can be mitigated during residency, which will lead to family medicine practitioners who can provide patient care.

## Methods

In-depth semi-structured interviews of 13 family medicine preceptors were undertaken at the largest IMG training sites in Canada to produce the original dataset. This method was utilized to collect data, as it was considered the most efficient method to gather opinions regarding observations in the clinical setting of this specific group of residents. Preceptors were recruited from a variety of positions, including: former program directors, current site directors, and community family physicians. Twenty-five preceptors were contacted via email to request their participation in the study, 13 consented to participate. Study participants’ gender ratio was nearly equal, including 7 males and 6 females (Table 1). All preceptors contacted had greater than 10 years of experience teaching, selecting or facilitating training of IMGs. Preceptors interviewed were from both rural and urban sites in the following programs: University of British Columbia (BC), University of Calgary (AB), Western University (ON), and University of Toronto (ON). These sites were selected as they are some of the largest national training sites with a high proportion of IMG trainees.

**Table 1:** Reference table regarding quotes in Table 3. Demonstrates that quotations presented are from a broad spectrum of sites and genders

Pseudo Identification Numbers	Province	Gender
Interv01	BC	F
Interv02	BC	F
Interv03	BC	M
Interv04	BC	M
Interv05	BC	M
Interv06	AB	F
Interv07	AB	M
Interv08	ON	F
Interv09	ON	F
Interv10	ON	F
Interv11	ON	M
Interv12	ON	M
Interv13	ON	M

Participants provided informed consent in writing prior to being interviewed and will remain anonymous for the purpose of this study. All interviews were conducted in person, at each interviewee’s teaching site, by the same interviewer based off an interview protocol to standardize data collection (Table 2). Preceptors were asked reflective questions, which provoked them to draw on examples of exceptional and poor IMG performers they have taught and selected. Interviewees were asked to share anecdotes to support their assertions and opinions. Human ethics approval from the Research Ethics Board (REB) was obtained from the University of British Columbia prior to conducting this qualitative descriptive study. Methods were performed in accordance with relevant guidelines and regulations designated by the REB. All interviews were audio-recorded then transcribed by a professional transcription company.

**Table 2:** Interview protocol and sample questions used to conduct semi-structured interviews for participants in the study

**Interview Protocol**	
***Introduction & Demographic Questions (Examples):*** ➔ What is your official title? ➔ How long have you been involved with this family medicine training site? ➔ Why did you choose to work in your current setting? ➔ What types of residents do you work with? Follow-up questions …	
***Selection Process Reflective Questions (Examples):*** ➔ What is your role in the selection of candidates? ➔ What factors in the selection process do you most heavily weigh upon? for IMGs and CMGs? ➔ What current selection tools are used at your institution? Follow-up questions …	
***IMG Experience (Examples):*** ➔ What is your role in the supervision of trainees? ➔ What is the number of IMGs you have supervised over the past 5 years? ➔ Can you share an example of an IMG candidate that thrived in residency? ➔ What attributes did that IMG candidate have that made them succeed? Follow-up questions …	
***Philosophy: Aims of selection (Examples):*** ➔ Do you think candidates should be chosen based on their adaptability? ➔ Do you think family physician training should be different depending on what type of practice they will be taking up in the future? Follow-up questions …	
***IMG Selection Philosophy Introspective Questions*** ➔ What do you value or discount in an IMG candidate’s application? ➔ Do you think we are missing something in the current selection process? Follow-up questions …	
***Closing Questions and follow up remarks***	

ATLAS.ti (9.1) qualitative software was used to code the data with standardized coding techniques in two tandem sequences. ANM and CD performed coding in two tandem sequences and LN performed verification of coding. Item analysis, pattern analysis and structural analysis were then used in three stages used to analyze the data [12]. This approach was justified to make sense of a large amount of data, which was organized into domains and subdomains. A code manager was used to separate the data into 14 domains and 101 subdomains. Analysis and collation of data revealed four major positive attribute domains. Each major code was then further predicted by up to 5 subcodes. Quotes were then collected through coding processes, which embodied each subcode. Each major code was confirmed to be an attribute and each subcode an identifiable predictor. The assumption was made that all communication by IMGs and CMGs with patients is in English as it is the dominant language in the provinces included in this study.

## Results

Coding of the data revealed four major attributes, which were most commonly associated with IMG residents’ success. These four attributes included: trainability, positive attitude, communication skills, and knowledge (Table 3). For each of the predictors, quotes were selected, which were determined to represent the general opinions of preceptors regarding each respective predictor (Table 3). Multiple subcodes were assembled as predictors of each major code (Table 4). All commonly presented predictors were determined to be useful attributes to assess in matriculation interviews.

**Table 3:** Major theme summary of positive attributes associated with an increased likelihood of residency success of IMG residents

Attribute	Predictors	Quote
**Trainability**	Professionalism	“Professionalism, always extremely professional [and in candidates we are’ looking for maturity, professionalism […] and reflectiveness.” (interv02)
		“We should be … recruiting doctors who are better nuanced in interpersonal skills.” (interv13)
		“[Unprofessional residents] in the classroom setting weren’t engaged, not interested, disruptive, … and on their phone.” (interv08)
	Ability to Accept and Integrate Feedback Well	“Their insight into their abilities, their lack of defensiveness when given feedback and to take feedback graciously and act on it.” (interv01)
		“The other thing which is harder to assess but really has to do with personal integrity, ability to take feedback, that whole professional package, right” (interv10)
		“[They] need to be able to improve [and be] willing to take feedback constructively.” (interv06)
	High Emotional Intelligence (Maturity)	“I value people who are truthful and who are genuine, or have a way to express their genuine beliefs” (interv13)
		“[How can you] state factors that you can’t read on a transcript or marks about the passion or the enthusiasm or the empathy that the candidate might have.” (interv07)
**Positive Attitude**	Presence of Positive Attitude	“I love having a resident with passion, loves to learn […] to me that’s huge, you’ve got to have that passion. Because there isn’t a day I don’t go home, 30 years later and start reading about something I saw in the office. If you don’t have that as a student or a resident, you wonder why they’re here.” (interv11)
		“The one who was very strong [they] just had a very go-get-em attitude.” (interv03)
**Communication Skills**	Proficient Communication Skills	“Fluency in English has different degrees. You can have fluency that you can travel and interact and go to the store, whereas to do an interview and take a sexual history or ask somebody about whether they’ve had any [delusions] – that requires a more sophisticated English.” (interv12)
		“One of the things that interests me [is] does the person have a breadth of activities that show collaboration and communication skills and ability to work with a team? Because I really think that more and more this is one of the most critical elements of all.” (interv04)
**Knowledge**	High Level of Medical Knowledge	“An IMG resident who was phenomenal and ended up winning, the outstanding resident award at the end of the two-year residency. [This resident …] has continued on to be a preceptor in the community and does amazing work. I think that … (this resident is a) medical expert and has a solid foundation and background.” (interv09)
		“[Of this program’s star IMG trainee] Let’s not even talk about the good medical training [he or she had], the [y had] superlative medical training […]” (interv05)

**Table 4:** Subcodes associated with positive attributes or an increased likelihood of residency success. Major themes are bolded at the top and examples to follow in the discussion italicized

	Major Codes			
	Attitude	Communication Skills	Knowledge	Trainability
SUBCODES	Leadership	Excellent Command of the English Language	Extensive Relevant Clinical Experience	Superior Ethics / Professionalism
	Passion for Learning	Fluency in a Broad Range of Canadian Language Colloquialisms	Superior Medical Knowledge	Teachability
	Positive Attitude		Recent Graduation	Extra Clinical Experience
	Honesty / Integrity			Ability to Accept and Integrate Feedback Well / Reflectiveness
	Team Player			High Emotional Intelligence (Maturity)

### Trainability - professionalism

Trainability was commonly present in the data associated with three predictors: professionalism, ability to accept and integrate feedback, and high emotional intelligence. The attribute that most commonly associated to an IMG resident’s trainability was professionalism. When asked why a certain trainee was succeeding, preceptors noted that the best candidates were *“always extremely professional [and in candidates we are] looking for maturity, professionalism [ …*] *and reflectiveness [interv02]”* (Table 3). Some interviewees asserted that professionalism was likely the most salient attribute of a candidate’s future success and if it was lacking, little can be done in mitigation of this shortcoming.

### Trainability - ability to accept and integrate feedback well

Trainability is further predicted by a resident’s ability to accept and integrate feedback. Ability to accept and integrate feedback is related to two main predictors, a resident’s teachability and reflectiveness. Family medicine preceptors communicated that excellent performers had *“insight into their abilities, [ …] lack [ed] defensiveness when given feedback and [took] feedback graciously and act [ed] on it [interv01]”* (Table 3*).* Thus, not only should a resident be able to accept feedback but must have the ability to integrate feedback and change their behaviours. It was also noted by family medicine educators in that, *“[residents] need to be able to improve [and be] willing to take feedback constructively [interv06]”* (Table 3). Residents’ who had the ability to accept feedback constructively were naturally more reflective and tended to improve much quicker than those lacking in this attribute.

### Trainability - high emotional intelligence

Interviewed preceptors indicated that they sought to evaluate emotional intelligence (EI) of applicants, as they attempted to identify genuine individuals who would demonstrate significant emotional maturity. *“[Preceptors] value people who are truthful and who are genuine, or [ …] express their genuine beliefs [interv13]”* (Table 3). Few preceptors were able to describe a reliable mechanism used to identify trainees with high EI at the time of selection. Preceptors expressed this challenge in determining EI as lacking the ability to *“[ …] state things that you can’t read on a transcript or [give] marks about the passion [ …] enthusiasm or empathy that the candidate might have [interv07]”* (Table 3). EI should be included in a list of attributes assessed, however should not replace quantitative attribute assessment such as clinical knowledge. EI was suggested as a differentiating attribute when assessing candidates of similar academic ability.

### Presence of positive attitude

Attitude was further predicted by the presence of leadership skills, a passion for learning, a positive attitude, honesty, and ability to be a team player. Interviewed preceptors noted the connection between positive personality traits and academic prowess with statements such as: *“the [resident] who was very strong, [ …] with respect to clinical knowledge, [ …] had a very go-get-em attitude [interv03]”* (Table 3). Thus, displaying the interconnectedness between personality and academic ability.

### Communication skills

Communication skills of a candidate were predicted by their ability to command the English language and having knowledge of a broad range of Canadian English colloquialisms. Excellent command of the English language was most commonly present in the context of the major code of communication skills. It would appear that family medicine preceptors felt that the degree of English language fluency can be a predictor of future success as *“fluency in English has different degrees. You can have fluency that you can travel and interact and go to the store, whereas to do an interview and take a sexual history or ask somebody about whether they’ve had any delusions – that requires a more sophisticated English [interv12]”* (Table 3). English fluency also relates to how proficient a candidate is at participating in collaboration in the workplace. For example, *“one [factor] that interests [preceptors is] if the [applicant has] a breadth of activities that show collaboration and communication skills and ability to work with a team, as [this is] one of the most critical things of all [interv04]”* (Table 3). Thus, preceptors indicated poor communication skills not only negatively impact direct patient care but the ability of a resident to participate in collaborative patient care efforts in the workplace.

### Clinical knowledge

Knowledge was predicted by three subcodes, of which superior medical knowledge was most commonly indicated to facilitate a selector’s ability to assess this attribute. An interviewee provided the example of *“an IMG resident who was phenomenal and ended up winning the outstanding resident award at the end of the two-year residency and [ …] has continued to [excel]. [This resident] is currently a preceptor for our program and continues to do amazing work. [This resident is truly a] medical expert, [with a] solid foundation and background [interv09]”* (Table 3). In addition, preceptors interviewed recalled that *“[their program’s star IMG trainee, had] superlative medical training [ …] [interv05]”* (Table 3). This rigor in training and knowledge was commonly associated with success in future certification examinations.

## Discussion

CanMEDS is a widely accepted framework to enhance physician training and this study elucidated predictors that support this model, with respect to its application to IMG selection and extend upon it with newer concepts [9]. It was found that trainability, positive attitude, communication skills, and knowledge are all positive predictors of IMG success in family medicine residency. These factors explain some holes in the literature with regards to why IMGs might be poor performers on examinations. In addition, these results shed light on weaknesses in the selection process and how we can work towards a more effective and holistic approach to selection of IMGs for family medicine residency.

### Trainability ➔ professionalism

Embedded in the definition of professionalism in the CanMEDS framework is a physician’s pledge to ongoing professional development, thus showing a strong link between a physician’s ongoing trainability and professionalism [9]. Professionalism was established as a positive predictor of IMG resident performance. However, it is recognized that professionalism can be a very difficult attribute to assess [13]. In addition, unprofessional residents present a strong association to lawsuits and adverse outcomes in their later careers [14]. Furthermore, unprofessional lapses in medical school are related to professionalism deficiencies in residency [13].

If a candidate lacks professionalism, their trainability is likely reduced and altering these qualities can prove very difficult. This is supported by the social psychology concept of ‘explanatory style’ [13]. It has been suggested that this can explain differences in people who incorporate versus discredit negative feedback [13]. Professionalism in medical education is an ongoing process that must be integrated throughout the curricula [15]. Thus, if a candidate has been educated in a system that lacks adequate professionalism development, it will be challenging to institute such changes so late in training. It has been suggested that if professionalism is assessed as lacking by a preceptor, this could indicate that a candidate may struggle in other competencies [3]. Therefore, it is recommended that professionalism is evaluated on checklists for assessment of IMG candidates.

### Trainability ➔ ability to accept and integrate feedback well

Teachability has been established as an important selection factor for residency programs and has close ties to a resident’s ability to accept feedback [16]. Ability to integrate feedback stems from a resident’s reflectiveness. Reflectiveness is an established positive predictor of a physician’s ability to improve and is a necessary attribute to successfully complete family medicine residency [17]. In addition, it has been demonstrated that measuring reflective capacity early in training positively correlates to clinical reasoning performance at the end of residency [18]. Thus, the ability to accept and integrate feedback are important factors to assess during selection. However, IMG residents come from a variety of educational backgrounds and are commonly at different stages in their lives than CMGs [11]. Therefore, it is important that each IMG candidate is carefully assessed in a diversity of contexts, as certain cultural norms may preclude proper assessment of this trait. Facilitators’ awareness and encouragement of thoughtful reflection can enhance a physician’s ability to integrate feedback [18]. Ability to accept and integrate feedback could be assessed in a situational judgement test (SJT) or a workplace-based interview where preceptors can assess a candidate’s response to specific situations.

### Trainability ➔ high emotional intelligence

Emotional Intelligence is understood as the ability to perceive, understand, and manage emotions in oneself and others [19]. Emotional intelligence levels have been presented as a potential non-academic predictor of success in healthcare settings [19]. EI should not be considered a replacement for cognitive intelligence assessment but may provide a method to differentiate candidates of similar cognitive ability. EI could be considered as an expansion of the CanMEDS role of collaborator, which is viewed as a physician’s ability to build relationships and their willingness to learn from others [9]. This suggests that a candidate with superior EI will be able to cultivate their role as a collaborator more proficiently and be more willing to learn, thus be more trainable, than a resident with less proficient EI. In addition, IMG residents who pass examinations on the first attempt tend to be strong collaborators and eager to build relationships to assist their learning [3].

It has been previously asserted that medical student selection should be based on both academic achievement and EI ability [19]. A physician’s EI capacity reflects their aptitude to enhance capacity in practice and enables them to better understand patient needs [20]. By assessing EI, it can reveal a resident’s ability to grow and learn in the future. However, this is a new and evolving concept not instituted in classical selection methods. EI is not explicitly part of the CanMEDS framework and is an emerging concept in the literature [9]. It has been proposed that SJTs can be used to assess EI along with other cognitive factors and could produce a more accurate estimate of future resident performance [21]. Additionally, workplace-based assessments could be used to assess EI along with a combination of selection criteria. However, due to the infancy of this concepts’ evolution an absolute method to assess this factor cannot be suggested. These results implore the need to expand on this concept and find ways to better assess and integrate EI into selection methods, as it is evident that EI cannot be used as a sole selection factor [21]. During the time of adaptability of this concept the need to assess its reliability and applicability is imperative to support this factor’s future use. By including EI in selection factors assessed in selection, this will allow for selection of candidates which encompasses a more holistic view of their future as a family medicine practitioner.

### Presence of positive attitude

Many medical educators have studied the correlation between personality and performance in an attempt to improve selection practices [22, 23]. The Big Five personality traits: extraversion, agreeableness, openness, conscientiousness, and neuroticism, have been deliberated extensively at the undergraduate admissions level and are linked with both strategic and deep learning [22]. In addition, personality at the time of selection has been associated with superior performance [23]. The assessment of a candidate’s attitude by selectors is critical as there are many barriers to a resident developing a personality fit for family medicine through teaching. Therefore, a positive attitude should be possessed by a candidate prior to matriculation. Assessing a resident’s personality can be very difficult in an interview setting [14]. The presence of a residents’ positive attitude should be assessed, however a working interview could prove to be more telling of this attribute. Selectors assessment of this trait can be complicated by the differences in the medical training that IMGs have received, thus these differences should be taken into account during assessment [11].

### Communication skills

High communication proficiency can act as a positive predictor for resident performance. A relationship between communication skills in English and clinical knowledge has been previously established [24]. The scope of communication should not be limited to that between a patient and clinician when selecting a candidate. A resident’s ability to communicate and collaborate with other members of a multidisciplinary team can facilitate their provision of holistic care and is a sign of future residency success. English is the dominant language spoken in the healthcare regions represented in this study, however it is important to note that a host of indigenous languages are also spoken in these areas. These pose an additional barrier to communication and cultural understanding for IMG residents. This provides an excellent opportunity to educate IMGs on cultural norms of indigenous populations, as they will not have the same knowledge as CMGs who have been educated in curricula that are striving to better serve indigenous populations in healthcare settings.

An IMG resident should be fluent in English to a level at which they can collaborate with others and detect subtle nuances in speech. This level of English fluency has been termed colloquial English and should be assessed [11]. IMG residents can frequently lack fluency in colloquial English, thus necessitating the importance of assessing this skill early on. Even if an IMG appears to be fluent in medical terminology in English, this should not be used as an indicator of their ability to converse colloquially [11]. If this attribute is assessed as inadequate during matriculation, IMG residents can be assisted in developing their English fluency, as ultimately their future diagnostic skills will be hindered if this shortcoming is not addressed. This displays the need for educators to be flexible when training IMG residents due to their varied backgrounds when entering family medicine residency [3].

### Clinical knowledge

It is widely accepted that possession of a superior level of clinical knowledge is associated with residency success and better performance on examinations [11]. However, a resident with superior clinical knowledge is not assured success on examinations without possession of other factors such as trainability [3]. Further explanation brings out the complexities of how clinical practice is clearly a continuum of systematic and critical assessment with experimentation and revision of knowledge. Studies have contemplated the roles of a physician and the very centre competency has been determined to be *medical expert* [9]. There is a high level of agreement among preceptors interviewed and the literature that residents who possess superior academic and clinical knowledge tend to excel in residency. Clinical knowledge is a strong predictor of future success but should be evaluated in conjunction with other factors [25]. It has been reported that IMGs who successfully passed exams on their first attempt had high levels of clinical knowledge in conjunction with valuing feedback [3]. In addition, teaching interpersonal competence can be as impactful on IMGs’ examination results as increasing their clinical knowledge [25].

Overall the methodology of this study is sound, however there are some limitations. The small sample size of preceptors interviewed introduces a sampling bias. This is a major limitation of the study, however through reproduction of results could be alleviated. Survivorship bias is evident, due to the method of results collection. The method did not facilitate determination of candidates possessing one or more of the predictors and still failing to perform. In addition, the applicability of results to other medical systems outside of Canada is limited as the group of preceptors interviewed did not have experience teaching in other medical systems. This study was carried out in regions of Canada where English is the dominant language when communicating with patients in healthcare settings, thus the results are situated within a Canadian context but all results are not directly generalizable to the rest of the country. In areas of data collection a host of indigenous languages are spoken but unfortunately not included in this study design, which is a both a limitation of this study and potential area for inclusion that future studies can strive to better represent in research. Determining a trainee’s ability to accept and integrate feedback and assess EI is another limitation of these results but could provide a mechanism to integrate these findings into selection processes.

## Conclusion

All identified predictors were determined to correspond with IMG family medicine residents’ success in residency. It has been previously well established that superior clinical knowledge correlates with a resident’s success [9, 11]. However, we sought to reproduce this finding, as well as elucidate other predictors of IMG success in the clinical environment. Presence of a positive attitude, communication skills, and trainability were all found to positively correlate with IMG residents’ success in family medicine. Assessing all of these factors consistently has yet to be determined in a concise method. SJTs, clinical simulations, and work-based interviews are all suggested methods to assess the breadth of candidates’ skills. With the implementation of these findings, expanded selection methods could be implored to facilitate more proficient evaluation of IMG candidates. Determining how the selection process should be supplemented for IMG and CMG candidates will continue to be a challenge. It is evident that the competencies revealed in this study are relevant to both IMGs and CMGs. However, how educational interventions are targeted to enhance culturally appropriate knowledge of interpersonal competencies is specific to enhancing IMG training. This is crucial in an international workforce that healthcare systems depend on, which underpins the importance of this study. Situational judgement tests, such as CASPer, have shown some development in this area, and could be used in adjunct for selection [26]. However, further studies regarding implementation into candidate selection have yet to be presented. Future studies regarding the common challenges IMGs face, and how they differ from CMGs, could elucidate innovations to assist in creation of a training environment to enhance IMG exam performance. Negative predictors of a residents’ success should be determined in future work and can be used in tandem with positive predictors to increase likelihood of selection of successful IMG residents. Overall, these results can provide a framework which sheds light on how to better, assess IMGs and place them in positions where they can succeed and ultimately maximize their benefit to patients.

## Data Availability

Not applicable.
