# Correction to: Challenges with international medical graduate selection: finding positive attributes predictive of success in family medicine residency

**DOI:** 10.1186/s12875-023-02058-w

**Published:** 2023-05-02

**Authors:** Alasdair Nazerali‑Maitland, Christina Douglas

**Affiliations:** 1 Department of Medicine, University of British Columbia, Diamond Healthcare Centre, 2775 Laurel Street, Vancouver, BC V5Z 1M9 Canada; 2 University of British Columbia, Vancouver, Canada

**Correction: BMC Primary Care 23:256 (2022)**

10.1186/s12875-022-01861-1

Following publication of the original article [1], the author Laura Nimmon should be removed from the authorship of the article.

Dr. Laura Nimmon is requesting removal of authorship. Based on the ICMJE criteria for author contributions Dr. Nimmon does meet the requirements for authorship. All authors have agreed to this change.

The authorship of this article and the original article has been corrected.
